# Chemical Composition and *in Vitro* Antifungal Activity Screening of the *Allium ursinum* L. (Liliaceae)

**DOI:** 10.3390/ijms13021426

**Published:** 2012-01-30

**Authors:** Radu Vasile Bagiu, Brigitha Vlaicu, Monica Butnariu

**Affiliations:** 1 Department of Hygiene, University of Medicine and Pharmacy “Victor Babes” 2A Eftimie Murgu Square, Timisoara 300041, Romania; E-Mails: bagiuradu@yahoo.com (R.V.B.); vlaicu@umft.ro (B.V.); 2 Chemistry and Vegetal Biochemistry, Banat’s University of Agricultural Sciences and Veterinary Medicine from Timisoara, Calea Aradului no. 119, Timisoara 300645, Romania

**Keywords:** ramsons, antifungal activity, extract, HPLC and GC/MS, MBC/MFC

## Abstract

The objective of the study was to summarize the methods for isolating and identifying natural sulfur compounds from *Allium ursinum* (ramson) and to discuss the active constituents with regard to antifungal action. Using chromatographic techniques, the active constituents were isolated and subsequently identified. Analyses by high-performance liquid chromatography (HPLC) suggested that these compounds were sulfur constituents, with a characteristic absorbance at 250 nm. Gas chromatography-mass spectrometry (GC-MS) analyses allowed the chemical structures of the isolated constituents to be postulated. We adopted the same methods to identify the health-giving profiling of ramsons and the effects are thought to be primarily derived from the presence and breakdown of the alk(en)ylcysteine sulphoxide, alliin and its subsequent breakdown to allicin (sulfur-compounds of ramson) in connection with antifungal action. The aim of the study was the characterization of the chemical composition of ramsons and the testing of the action of the *in vitro* extracts, on different strains of *Candida albicans*. The main goal was to highlight the most efficient extracts of *Allium ursinum* that can provide long-term antifungal activity without remissions. The extracts from *Allium ursinum* plants, inhibited growth of *Candida* spp. cells at concentrations ranging from 0.5 to 4.0 mg/mL, while that of adherent cells at concentrations ranging from 1.0 to > 4.0 mg/mL, depending on the yeast and plant species.

## 1. Introduction

In recent years there has been an increased interest in the use of natural compounds, and questions concerning the safety of synthetic compounds have encouraged more detailed studies of plant resources. Sulfur compounds, the extracts, the odours and volatile products of plant secondary metabolism, have a wide application in folk medicine, food flavouring and preservation as well as in the fragrance industry. The antifungal properties of sulfur compounds have been known for many centuries. The use of plant extracts is in general an alternative form of therapy for mycosis and candidiasis [1]. Literature data showed that there are directly proportional relationships between the type and concentration of the sulfur from *A. ursinum* and antimicrobial action of these plants [2].

The main cysteine sulfoxides were alliin and isoalliin. It has been found that alliinase of *A. ursinum* exhibited properties similar to those of alliinase of garlic (*Allium sativum* L.), but differed in terms of substrate specificity.

The reason plants synthesize these compounds is to defend against microscopic pests (fungi or viruses) or macroscopic (insects) [3]. The action mechanism involves the biosynthesis of some elicitors (molecules of immune defence and synthesized toxins with lethal effect on pests). In selecting plants and types of extracts with antifungal effect, we chose to investigate *A. ursinum*.

Opportunistic infections of fungal etymology are part of the emerging infectious diseases [4] and are becoming an increasing proportion, especially in the context of synthetic antibiotics abuse [5]. The severity of infections caused by *Candida* is determined by the balance between the pathogenicity of the microorganism and the host’s defence mechanism and involves damage to the immune system [6]. Genus *Candida* includes yeast fungi-forms which reproduce by budding and forming blastospores. *Candida albicans* is present in approximately 50% of the population without causing signs or symptoms of disease and is found at different levels in the oral cavity, vagina, and gastrointestinal tract. These areas may be, under the influence of a variety of factors; true “reservoirs” of *Candida albicans*, speeding up the emergence of events. These factors include: prolonged use of antibiotics, changes occurring during pregnancy, menstruation and menopause as well as following hormonal therapy, including use of contraceptives [3], together with other infection factors or associated diseases, deficiencies of the immune system and diabetes [7–9]. Antifungal allopathic medication includes various substances such as hexoral, fungizon, ampfocin, fluconazole, miconazole, *etc*. [10].

Given the multitude of bioactive compounds in *A. ursinum*, particularly of extracts of sulfur compounds that are known to have antimicrobial potential, we aimed to characterize the chemical composition of ramsons and the relationship of these parameters to the antifungal activity tested *in vitro* on strains of *Candida* [11–13]. The most familiar or important chemical constituents reported from ramsons are the sulfur compounds. It has been estimated that cysteine sulfoxides (alliin) and the non-volatile γ-glutamylcysteine peptides make up more than 82% of the total sulfur content of ramsons [14]. The thiosulfinates, ajoenes—a degraded form of allicin—vinyldithiins and sulfides, however, are not naturally occurring compounds [15]. Thiosulfinates formed in *Allium* are degraded to various polysulfides and ajoenes which also exhibit different degrees of antimicrobial activity [12]. The volatile sulfur compounds show more potent inhibitory effects towards fungi than bacteria. To some extent, they are degradation products from the naturally occurring cysteine sulfoxide, alliin. When the bulb of the ramson is crushed, minced, or otherwise processed, alliin is released from divisions and interacts with the enzyme alliinase in adjacent vacuoles. Hydrolysis and condensation of the reactive intermediate (allylsulfenic acid) forms allicin [16].

Allicin itself is an unstable product and undergoes additional reactions to form other derivatives (products), depending on environmental and processing conditions [17]. Extraction of leaves of ramson with ethanol at <0 °C gave alliin; extraction with ethanol and water at 25 °C led to allicin and no alliin; and steam distillation converted the alliin totally to diallyl sulfides [18]. The content of alliin was also affected by the processing treatment: leaves of ramson (fresh) contained 0.25–1.15% alliin, while material carefully dried under mild conditions contained 0.7–1.7% alliin. Gamma-glutamylcysteine peptides are not acted on by alliinase. On prolonged storage or during germination, these peptides are acted on by γ-glutamyltranspeptidase to form thiosulfinates [19]. This study aims to show that plants of *A. ursinum* can be used in different antifungal formulations. Qualitative and quantitative assay of the content of sulfur constituents (alliin, allicin *etc*.) were studied by means of HPLC or GC/MS methods.

## 2. Results and Discussion

### 2.1. Identification of Allicin from *Allium ursinum* Extract

The identification of allicin and some derived compounds from fresh *Allium ursinum* was carried out. Two compounds were extracted from the leaves of fresh ramsons. The first was identified as allicin, which is a very unstable compound at room temperature. Taking advantage of this instability, the thermal degradation of the obtained allicin was induced in order to form a mixture of volatile compounds.

Allicin was isolated using a chromatographic method with an HPLC instrument.

### 2.2. Gas Chromatography Separates the Components of *Allium ursinum* Extract

Gas chromatography is used to separate the components of a mixture while mass spectroscopy can then characterize each of the components individually. Problems, when using these methods, may occur because of the release and activity of the above mentioned compounds during material preparation, as this might change the composition of the material to be analysed. The most abundant of the volatile compounds from *A. ursinum* were diallyl disfulfide (19.98), diallyl trisulfide (38.74), 3-vinyl-(4*H*)-ditiin-1,2 (42.90) and 2-vinyl-(3*H*)-1,3-ditiin (57.87). These compounds and their derivatives from ramsons, have been reported by other authors [20].

### 2.3. GC/MS of Volatile Compounds from *S*-methyl Cysteine Sulfoxide from *A. ursinum* Extract

This concerns sulfur compounds that are involved in multiple biological activities of plants. Both allicin and volatile compounds were evaluated in the leaves of ramsons. The second non-volatile compound obtained in this work was identified as *S*-methyl cysteine sulfoxide, the pattern of mass fragmentation coinciding with what was indicated in the literature for this compound which showed the molecular ion of 151 DA [21]. In the mix of volatiles generated from S-methyl cysteine sulfoxide, three compounds of greater concentration were identified: dimethyl disulfide (11.31), dimethyl trisulfide (9.24) and dimethyl tiosulfonate (16.49). Detection of active constituents was obtained, with ions at *m/z* (449 + 451) > *m*/*z* (269; 271; 287; 289), which are specific to natural sulfur compounds. It was observed that allicin underwent complete decomposition at 20 °C after 20 h resulting in diallyl disulfide (DADS), diallyl trisulfide, diallyl sulfide and sulfur dioxide. However, allicin underwent complete decomposition at 40 °C after 144 h. The particular instability of the allyl compound appears to be associated with the double bond [22].

### 2.4. Efficacy of Antifungal of *Allium ursinum* Extracts

This concerns compounds as precursors for sulphur (*S*-) in vegetables of the genus *Brassica* and *Allium*.

The efficacy and safety of antifungal drugs depends on: their spectrum of activity, their actions, the minimum fungicidal concentration, and the minimal inhibitory concentration.

Pharmacologically, allicin is the most important and the most active substance and it is found in the fresh extract of leaves of the ramson. The mechanism of the action of sulfur compounds towards microorganisms is complex and has not yet been fully explained. It is generally recognised that the antimicrobial action of sulfur compounds depends on their hydrophilic or lipophilic character.

**Figure 1:**
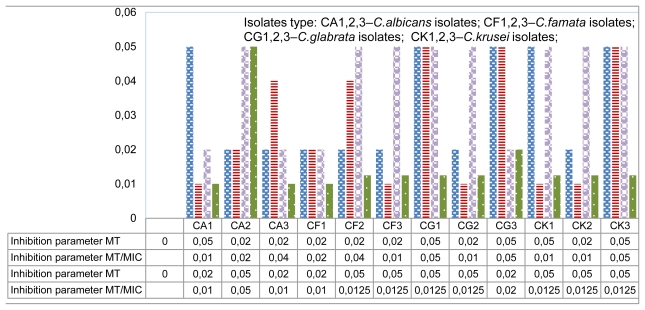
The morphogenetic transformation (MT) values and MT/MIC ratios.

The results of the antibacterial activity assays of sulfur compounds on different sources of *S*-methyl cysteine sulfoxide isolated and identified in this work, as well as the mixes of volatiles generated by these two compounds, indicated that this activity was strong. In antifungal investigations we used the macro- and micro-dilution method in order to determine minimum inhibitory concentration (MIC) and minimum fungicidal concentration (MFC) values. MIC values in microdilution assay were 0.5–4.0 μL/mL, the same as in macro-dilution assay 1.0–4.0 μL/mL with a low ratio of MFC/MIC value 1.0 or 2.0. Thus, we can conclude that linalool possesses strong antifungal activity [23,24]. These ratios can be used to determine the relative potency of each agent to inhibit growth and transformation: ratio <1 agents which preferentially inhibit morphogenetic transformation; ratio of 1–2 those with approximately equal effects on these two processes; and ratio >2 agents with a lower ability to inhibit the hyphal form but which exert their activity by inhibiting the yeast form. In order to describe the differences better, the morphogenetic transformation/MIC ratios were calculated for each antifungal agent (Table 1 and Figure 1). MIC and morphogenetic transformation (MT) values are expressed as the median values (mg/L) for each antifungal agent. The values are the mean of at least three determinations. The median was calculated only on the basis of the strains susceptible, as judged by MIC; those with reduced susceptibility or resistant to drugs were excluded.

The data suggest that agents with a high fungicidal potential, also have a high potential to block morphogenetic transformation. Agents with a low fungicidal potential are strikingly less able to inhibit morphogenetic transformation [25]. Certain sulfur compounds of leaves of ramson extract can act as uncouplers, which interfere with proton translocation over a membrane vesicle and subsequently interrupt ADP phosphorylation (primary energy metabolism). Specific natural sulfur compounds with functional groups, such as phenolic alcohols or aldehydes [26], also interfere with membrane–integrated or associated enzyme proteins, stopping their production or activity.

Sulfur compounds also inhibit the synthesis of DNA, RNA, proteins and polysaccharides in fungal and bacterial cells. In fungi, they evoke changes similar to the effects of antibiotic action [27].

## 3. Experimental Section

### 3.1. Vegetal Material

*Allium ursinum* was collected in Didactics resort (Western part of Romania) of the Agricultural Faculty, Banat’s University, in blossoming phase. The Voucher Herbarium specimen is deposited in the Department of Biology. The experiments were carried out in the Vegetal Biochemistry Laboratory of the Banat’s University and Department of Hygiene.

### 3.2. Obtaining and Evaluating *Allium ursinum* Extract

Allicin was obtained from 5 g of fresh leaves of ramson, homogenized in 30 mL of distilled water for 5 min at 4 °C. The result was mixed and incubated at 30 °C for 20 min. Then it was centrifuged for 20 min at 4 °C, after which 1.5 mL of methanol was added, the centrifugation operation was repeated for 5 min at 4 °C. Allicin was isolated using a chromatographic method with an HPLC instrument. An HPLC instrument Shimadzu liquid chromatography with an LC-10 ADVP pump, UV/VIS detector was used. The peaks were recorded in a C-R8A Chromatopac Integrator. Aliquots of ramson extract and the fractions were separately filtered through 0.45 μm syringe filters before injection into the HPLC column. The HPLC mobile phase was prepared by combining equal volumes of methanol and distilled water (formic acid); the mobile phase was degassed in an ultrasonic bath for 30 min. The HPLC conditions were as follows: column temperature, 28 °C; 250 nm; flow rate, 0.8 ml min^−1^; sample, 1 μL; run time, 15 min; attenuation, 2; chart speed, 2 mm/min. Chemical product identification was based on information related to the retention time in HPLC of 6.5 min, mass fragmentation and the ultraviolet spectrum, which coincided with the characteristics of a standard, prepared at 10 mg/mL [20].

### 3.3. GC/MS Analysis of the Volatile Compounds

5 mL of allicin (identified by HPLC) were left in a tightly closed bottle at room temperature for 72 h.

The generated volatile compounds were recovered and identified using the chromatographic method. The prepared ramson plant extract was subjected to GC/MS analysis using Shimadzu GC/MS–QP 5050 A. Software Class 5000. Column: DB5, 30 m, 0.53 mm ID, 1.5 μm film. Carrier gas: Helium (flow rate 1 mL/min.). Ionization mode: EL (70 eV). Temperature program: 30 °C (static for 2 min) then gradually increasing (100 °C at a rate of 2 °C/min) up to 150 °C (static for 7.5 min).

Detector temperature was 150 °C and Injector temperature was 150 °C. The samples were detected in scan and in SIM modes. The retention time of allicin in the above described conditions was 0.9 min.

Purity was continuously checked by the control of the ratio between the identifying ion and another ion, specific for each compound. As already presented, the concentration for each compound in the biological samples was calculated from the ratio between the area of the organosulfur compound and the product of the internal standard for extraction and the area of the internal standard for final volume [22].

### 3.4. Obtaining and Evaluating the S-methyl Cysteine Sulfoxide

*S*-methyl cysteine sulfoxide was made from 200 g of fresh leaves of ramson mixed with methanol 95% for three days at room temperature, followed by additional mixing. The next step was to filter through a Whitman 4 paper filter. The extract was concentrated in a rotary evaporator. The obtained extract was separated in a chromatographic column filled with a cationic resin (AG–50WX2), the fraction containing *S*-methyl Cysteine sulfoxide was separated with 1 N NH_4_OH (ammonium hydroxide). The fraction was concentrated and *S*-methyl Cysteine sulfoxide was purified and identified by HPLC, using the method described for obtaining allicin (used column was Lichrosphere 5RP–18 25 × 10, 5 μ). An isocratic method was used with a mobile phase of 16% of acetonitrile in a buffer zone of 50 mM potassium phosphate (pH 7.0). Detection was made at 337 nm.

### 3.5. Obtaining and Evaluating the *S-*methyl Cysteine Sulfoxide Volatile

*S*-methyl cysteine sulfoxide obtained as described in the previous paragraph was thermally degraded in an autoclave at 121 °C for 15 min. Volatile compounds generated were recovered and identified in the same way as allicin. The oven temperature from 40 to 200 °C increases at 2.4 °C/min.

Injection and detector temperatures were 250 and 280 °C; ion source 70 eV worked in the mass range 40–200 Da [22].

### 3.6. Determination of Minimum Inhibitory Concentration (MIC) and Minimum Bactericidal/Fungicidal Concentration (MBC/MFC)

Minimum inhibitory concentration (MIC) was determined for the leaves of ramson extract and it showed antimicrobial activity against test pathogens [28]. The micro dilution method was followed for the determination of MIC values. *A. ursinum* extracts were suspended in acetone (which has no activity against test microorganisms) to make 10 mg/mL final concentration, then two fold serially diluted; and added to broth media of 96-wells of microtiter plates. Thereafter 100 μL inoculum (for bacteria 1 × 10^8^ colony-forming unit (CFU)/mL and 1 × 10^7^ cell/mL for yeast) was added to each well (10 μL samples were removed from all wells). Bacterial and fungal suspensions were used as the negative control, while broth containing standard drug was used as thepositive control. The microtiter plates were incubated at 37 °C for 24 h for bacteria and 28 °C for 48 h for yeast. Each extract was assayed in triplicate and each time two sets of microplates were prepared, one was kept for incubation while the other set was kept at 4 °C for comparing the turbidity in the wells of the microplate. The MIC values were taken as the lowest concentration of the extracts in the well of the microtiter plate that showed no turbidity after incubation. The turbidity of the wells in the microtiter plate was interpreted as visible growth of microorganisms. The minimum bactericidal/fungicidal concentration (MBC/MFC) was determined by sub-culturing 50 μL from each well showing no apparent growth. The least concentration of extract showing no visible growth on sub culturing was taken as MBC/MFC [29].

### 3.7. Determination of MICs and Morphogenetic Transformation

Synchronized yeast-phase *C. albicans* cells were used in all experiments and were prepared as described previously. A total 9 strains of C*andida* spp., obtained from the nasopharynx of patients, included *C. albicans* (3 isolates), *C. famata* (3 isolates), *C. glabrata* (3 isolates), *C. krusei* (3 isolates) and were used throughout. The isolates were stored at −20 °C in 50% glycerol and cultured on Sabouraud dextrose agar at 30 °C for 48 h; before each experiment, the isolates were subcultured on Sabouraud glucose broth (further called Sabouraud medium) at 30 °C for 48 h. Stationary phase synchronized yeast cells were harvested by centrifugation, washed three times in 0.1 M phosphate-buffered saline and used immediately in standard MIC assays and morphogenetic transformation experiments. The effects of different antifungal agents on the morphogenetic transformation of *C. albicans* were assessed by examining the contents of 96-well microtitre plates after 3 h incubation at 35 °C using phase-contrast microscopy (using an Olympus CK microscope and Zeiss phase-contrast microscope) [30].

### 3.8. Minimum Fungicidal Concentration

Samples (10 μL) were removed from all wells of the standard MIC plates and spotted on to rectangular dishes containing Sabouraud dextrose agar. The plates were incubated for 24–48 h at 35 °C.

The minimal fungicidal concentration (MFC) was defined as the concentration of antifungal agent at which the number of colony forming units was zero [28].

### 3.9. Antifungal Agents/Chemicals

All chemicals used were of analytical purity from Merck and Sigma (methanol, formic acid, allicin standard, acetone, *etc*.). Antifungal agents were dissolved in dimethyl sulfoxide (DMSO)/sterile distilled water. DMSO (final concentration of <2% v/v) did not affect the MIC or morphogenetic transformation.

### 3.10. Statistical Analysis

The values are the mean of at least three determinations. The median was calculated only on the basis of the strains susceptible as judged by MIC; those with reduced susceptibility or resistant to drugs were excluded.

## 4. Conclusions

The aim of this paper was to compare the activity of the extract of *Allium ursinum*, against cells of *Candida* spp. (*C. albicans*, *C. famata*, *C. glabrata* and *C. krusei*).

The extracts from *Allium ursinum* plants, inhibited growth of cells of *Candida* spp. at concentrations ranging from 0.5 to 4.0 mg/mL, while that of adherent cells at concentrations ranging from 1.0 to >4.0 mg/mL, depending on the yeast and plant species.

From the results obtained it is possible to conclude that the allicin and *S*-methyl cysteine sulfoxide isolated and identified in this work, as well as the mixes of volatiles generated by these two compounds, were capable of inducing antifungal activity.

In conclusion, the morphogenetic transformation and MIC assays used in this study, combined with the calculation of morphogenetic transformation/MIC ratios, may assist in screening for antifungal agents. The morphogenetic transformation assay would greatly assist in the characterization of the activity of new antifungal agents and may help to distinguish fungicidal from fungistatic compounds.

The assay is rapid: data are generated within 3 h, as compared with 48 h for standard MIC procedures. In this work the volatile compounds (natural sulfur compounds) induced a greater percentage of bud breaking than the non-volatile compounds.

In summary, the results indicate that some of the natural sulfur compounds exhibited promising fungistatic activities and they warrant more consideration as prospective antimicrobials.

There are limitations also in the use of animal models; this study highlights the need for an improved animal model.

## Figures and Tables

**Table 1:** *In vitro* activity *A. ursinum* extract of against cells of *Candida* spp.

Isolates type	Inhibition parameter					

	MIC (mg/mL)	MFC (mg/mL)	MFC/MIC	MIC (mg/mL)	MFC (mg/mL)	MFC/MIC

	*A. ursinum* extract			Volatile compounds of *A. ursinum* extract		
CA_1_	0.5	1.0	2.0	2.0	2.0	1.0
CA_2_	1.0	1.0	1.0	1.0	2.0	2.0
CA_3_	0.5	0.5	1.0	2.0	2.0	1.0
CF_1_	1.0	1.0	1.0	2.0	2.0	1.0
CF_2_	0.5	0.5	1.0	4.0	4.0	1.0
CF_3_	2.0	2.0	1.0	4.0	4.0	1.0
CG_1_	1.0	2.0	2.0	4.0	4.0	1.0
CG_2_	2.0	2.0	1.0	4.0	4.0	1.0
CG_3_	1.0	2.0	2.0	1.0	1.0	1.0
CK_1_	0.5	1.0	2.0	4.0	4.0	1.0
CK_2_	2.0	2.0	1.0	4.0	4.0	1.0
CK_3_	0.5	1.0	2.0	4.0	4.0	1.0

CA_1,2,3_—*C. albicans* isolates; CF_1,2,3_—*C. famata* isolates; CG_1,2,3_—*C. glabrata* isolates; CK_1,2,3_—*C. krusei* isolates; The values are the mean of at least three determinations. MIC—minimum inhibitory concentration and MFC— minimum fungicidal concentration.
